# Metabolite labelling reveals hierarchies in *Clostridium acetobutylicum* that selectively channel carbons from sugar mixtures towards biofuel precursors

**DOI:** 10.1111/1751-7915.12459

**Published:** 2016-11-22

**Authors:** Ludmilla Aristilde

**Affiliations:** ^1^ Department of Biological and Environmental EngineeringCollege of Agriculture and Life SciencesCornell UniversityIthacaNY14853USA

## Abstract

Clostridial fermentation of cellulose and hemicellulose relies on the cellular physiology controlling the metabolism of the cellulosic hexose sugar (glucose) with respect to the hemicellulosic pentose sugars (xylose and arabinose) and the hemicellulosic hexose sugars (galactose and mannose). Here, liquid chromatography–mass spectrometry and stable isotope tracers in *Clostridium acetobutylicum* were applied to investigate the metabolic hierarchy of glucose relative to the different hemicellulosic sugars towards two important biofuel precursors, acetyl‐coenzyme A and butyryl‐coenzyme A. The findings revealed constitutive metabolic hierarchies in *C. acetobutylicum* that facilitate (i) selective investment of hemicellulosic pentoses towards ribonucleotide biosynthesis without substantial investment into biofuel production and (ii) selective contribution of hemicellulosic hexoses through the glycolytic pathway towards biofuel precursors. Long‐term isotopic enrichment demonstrated incorporation of both pentose sugars into pentose‐phosphates and ribonucleotides in the presence of glucose. Kinetic labelling data, however, showed that xylose was not routed towards the biofuel precursors but there was minor contribution from arabinose. Glucose hierarchy over the hemicellulosic hexoses was substrate‐dependent. Kinetic labelling of hexose‐phosphates and triose‐phosphates indicated that mannose was assimilated but not galactose. Labelling of both biofuel precursors confirmed this metabolic preference. These results highlight important metabolic considerations in the accounting of clostridial mixed‐sugar utilization.

## Introduction

Decomposition of lignocellulosic wastes by anaerobic bacteria, including the *Clostridium* species, is an important component in the turnover of organic carbons in soils. Several of the soil‐dwelling *Clostridium* species, including notably *Clostridium acetobutylicum*, have been exploited for biofuel production due to their ability to ferment sugars from polysaccharides and produce hydrogen gas, short‐chain carboxylic acids (butyrate, acetate), alcohols (ethanol and butanol) and ketones (acetone) (Grupe and Gottschalk, 1992; Dürre, 1998; Desai *et al*., 1999; Gheshlagi *et al*., 2009; Ren *et al*., 2010; Amador‐Noguez *et al*., 2011; Hu *et al*., 2011; Servinsky *et al*., 2012; Aristilde *et al*., 2015; Dash *et al*., 2016). Polysaccharides from lignocellulosic wastes are composed of a mixture of different types of sugars, which include primarily the following: the hexose glucose from cellulose, the pentoses xylose and arabinose from hemicellulose, and other hexoses (mannose, galactose, in addition to glucose) from hemicellulose. The different sugars in the hemicellulosic component are dependent on the source material (Scheller and Ulvskov, 2010). Of particular interest for optimizing the conversion of plant waste materials to biofuel products in clostridial species is a comprehensive understanding of the cellular metabolism of glucose with respect to hemicellulosic hexoses and pentoses.

**Figure 1:**
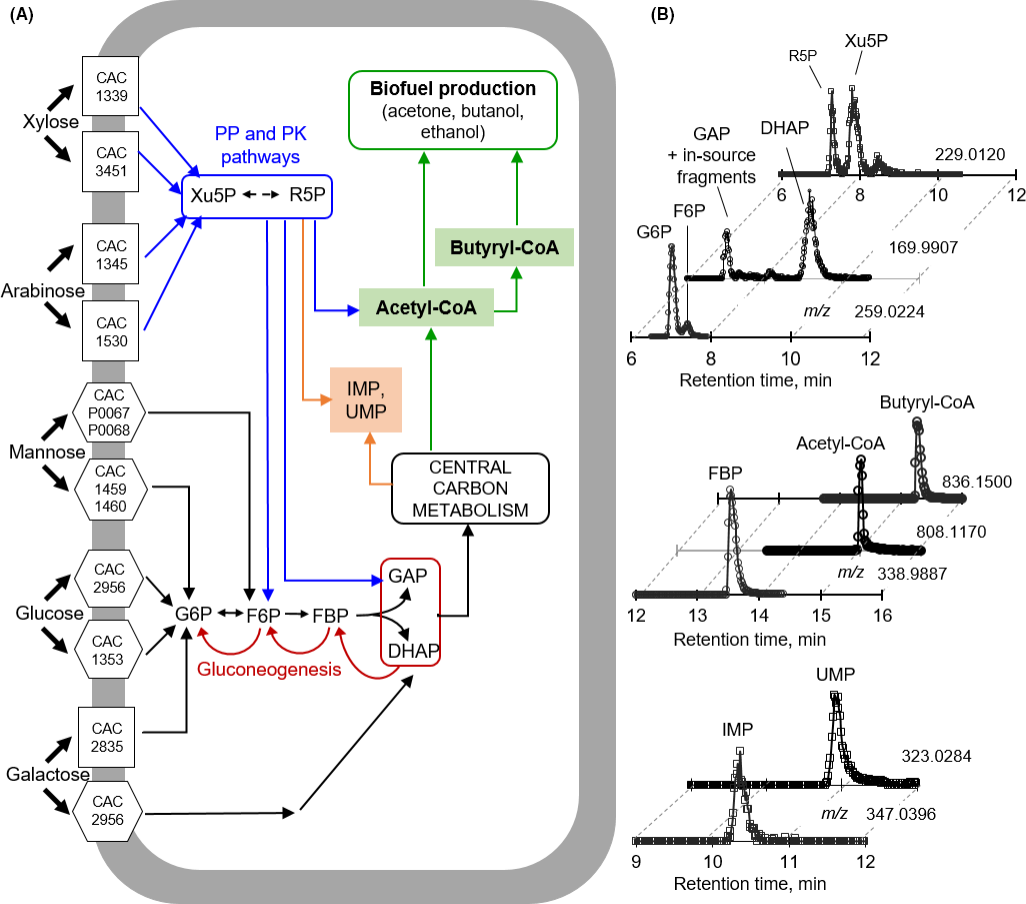
Schematic central carbon metabolism and metabolite identification. (A) Schematic routing of sugar catabolism towards biofuel production following sugar uptake in *Clostridium acetobutylicum*. The black lines represent catabolic pathways for the hexose sugars and in the central carbon metabolism, the blue lines represent catabolic pathways of pentose sugars through the pentose‐phosphate (PP) and phosphoketolase (PK) pathways, the dark red lines show reactions involved in gluconeogenesis; the genes reported to encode the sugar uptake transporters are shown (Servinsky *et al*., 2010). (B) Liquid chromatography–mass spectrometry chromatogram of important metabolites isolated from glucose‐grown cells. Abbreviations: G6P, glucose 6‐phosphate; F6P, fructose‐6‐phosphate; DHAP, dihydroxyacetone‐phosphate; GAP, glyceraldehyde‐3‐phosphate; Xu5P, xylulose‐5‐phosphate; R5P, ribose‐5‐phosphate; FBP, fructose‐1,6‐bisphosphate; Acetyl‐CoA, acetyl‐coenzyme A; butyryl‐CoA, butyryl‐coenzyme A; IMP, inosine monophosphate; UMP, uridine monophosphate.

Transcriptional analysis of *C. acetobutylicum* grown on each hexose or pentose sugar as a sole carbon source revealed the expression of all the relevant sugar uptake transporters (Servinsky *et al*., 2010; Mitchell, 2016; Fig. 1A). In accordance with these findings, growth of *C*. *acetobutylicum* on each sugar as the single carbon source was reported for glucose (Amador‐Noguez *et al*., 2011; Aristilde *et al*., 2015), galactose (Guiterrez and Maddox, 1996; Raganati *et al*., 2012), mannose (Raganati *et al*., 2012; Voigt *et al*., 2014), xylose (Raganati *et al*., 2012; Aristilde *et al*., 2015; Kudahettige‐Nilsson *et al*., 2015) and arabinose (Raganati *et al*., 2012; Servinsky *et al*., 2012; Zhang *et al*., 2012; Aristilde *et al*., 2015). The genes encoding for the two mannose transporters were shown to be expressed within the same order of magnitude during growth on mannose alone versus glucose alone, with slightly higher expression during growth on mannose alone (Servinsky *et al*., 2010; Fig. 1A). By contrast, the corresponding genes for galactose transport were completely suppressed when cells were grown on glucose alone (Servinsky *et al*., 2010; Fig. 1A). The genes encoding the four uptake transporters, two each for xylose and arabinose, were highly expressed in cells grown on xylose alone or arabinose alone but not in glucose‐grown cells (Servinsky *et al*., 2010). Thus, these transcriptional results implied that glucose‐grown *C. acetobutylicum* may accommodate uptake of mannose but not of galactose, xylose or arabinose. However, much still remains unknown regarding the simultaneous utilization of both glucose and another sugar in *C. acetobutylicum*.

Following uptake, pentose and hexose sugars follow distinct paths through the central carbon metabolism and towards two important biofuel precursors: acetyl‐coenzyme A (acetyl‐CoA) and butyryl‐coenzyme A (butyryl‐CoA) (Fig. 1A). Acetyl‐CoA is a precursor to acetate and ethanol (Lee *et al*., 2008; Aristilde *et al*., 2015; Dash *et al*., 2016). Acetyl‐CoA combines with another acetyl‐CoA to produce acetoacetyl‐CoA, which is a precursor to acetone and butyryl‐CoA; the latter is used to synthesize butyrate and butanol (Lee *et al*., 2008; Aristilde *et al*., 2015; Dash *et al*., 2016). Glucose, the primary hexose, is metabolized through the glycolytic pathway, which involves glucose phosphorylation to hexose‐phosphates [glucose 6‐phosphate (G6P) and fructose‐6‐phosphate (F6P)] followed by splitting of a bis‐phosphorylated hexose, fructose‐1,6‐bisphosphate (FBP), to triose‐phosphates [glyceraldehyde‐3‐phosphate (GAP) and dihydroxyacetone‐3‐phosphate (DHAP)] (Fig. 1A). These triose‐phosphates subsequently feed into the central carbon metabolism, which connects to the production of biofuels (Fig. 1A).

Mannose‐derived carbons can enter metabolism either via G6P, similar to glucose, or via F6P (Servinsky *et al*., 2010); galactose‐derived carbons are catabolized either via the Leloir pathway that generates G6P or the tagatose‐6P pathway that directly produces GAP and DHAP (Sund *et al*., 2013; Fig. 1A). The gene for the enzyme that connects phosphorylated mannose to glycolysis via F6P was similarly expressed in both glucose‐grown and mannose‐grown *C*. *acetobutylicum* (Servinsky *et al*., 2010). On the other hand, the genes responsible for galactose assimilation were minimally expressed when cells were grown on glucose alone (Servinsky *et al*., 2010) (Fig. 1A). Preferential consumption of glucose over galactose in a *C. acetobutylicum* during growth on both substrates implied that inhibition of galactose assimilation by the presence of glucose may persist even when galactose is also present (Guiterrez and Maddox, 1996). It is not yet clear how mannose transport and assimilation are influenced by the presence of glucose.

With respect to pentose sugar metabolism, there are two paths for their catabolic route to biofuel production. Following phosphorylation to xylulose‐5‐phosphate (Xu5P) and ribose‐5‐phosphate (R5P), pentose sugars get fed into the pentose‐phosphate (PP) pathway, which connects to the glycolytic pathway by generating the hexose‐phosphate F6P and the triose‐phosphate GAP. Alternatively, metabolites in the PP pathway can bypass most of glycolysis to produce acetyl‐CoA directly through the phosphoketolase (PK) pathway (Fig. 1A; Servinsky *et al*., 2012; Liu *et al*., 2012; Aristilde *et al*., 2015). The PK reactions involve the cleavage of the PP pathway metabolite Xu5P or the glycolytic metabolite F6P to produce, respectively, GAP and acetyl‐phosphate (acetyl‐P) or erythrose‐4‐phosphate and acetyl‐P. The metabolite acetyl‐P can be converted directly to either acetate or acetyl‐CoA. Genes involved in pentose catabolism in both the PP and PK pathways were found to be still expressed in glucose‐grown cells, though less abundantly (Servinsky *et al*., 2010, 2012). The inhibition of pentose metabolism in the presence of glucose has been well documented in *C. acetobutylicum* (Ounine *et al*., 1985; Fond *et al*., 1986; Gu *et al*., 2009; Grimmler *et al*., 2010; Aristilde *et al*., 2015). Despite the seemingly minimal pentose utilization in *C. acetobutylicum* in the presence of glucose based on extracellular substrate depletion, ^13^C‐labelling experiments recently revealed that assimilated pentose carbons from glucose:pentose mixtures can be accumulated in PP pathway intermediates, leaving glucose as the dominant sugar incorporated into glycolytic metabolites (Fig. 1A; Aristilde *et al*., 2015). Regarding the involvement of the PK pathway, the labelling patterns of acetyl‐P in tracer experiments revealed an increased participation of the PK pathway in the presence of arabinose whereby arabinose‐derived carbons were routed through the PK pathway during feeding on glucose:arabinose mixture (Aristilde *et al*., 2015). Whether the PK pathway may also provide a connection between arabinose catabolism and biofuel precursors in the presence of glucose was not determined.

In addition to connecting assimilated pentoses to the glycolytic and PK pathways, the PP pathway provides the ribose sugar backbone for inosine monophosphate (IMP) and uridine monophosphate (UMP). These two metabolites are required for *de novo* ribonucleotide biosynthesis: IMP is a precursor to purines and UMP is a precursor to pyrimidines (Fig. 1A). Pentose accumulation in the PP pathway in the presence of glucose was thus proposed to serve as a strategy to route pentoses towards ribonucleotide synthesis during growth on glucose:pentose mixtures (Aristilde *et al*., 2015). Confirmation of this metabolic strategy has not yet been reported in *C. acetobutylicum* or other clostridium species.

Building on the aforementioned studies, this study employs a metabolomics approach to investigate the following four hypotheses regarding the co‐metabolism of glucose and each hemicellulosic sugar in *C. acetobutylicum*: (1) glucose inhibits galactose metabolism and its subsequent contribution to biofuel precursors; (2) glucose does not compromise mannose metabolism towards biofuel precursors, (3) pentoses are routed from the PP pathway towards ribonucleotide biosynthesis but not glycolysis, and (4) pentoses are routed from the PP pathway to biofuel precursors through the PK pathway in the presence of glucose. High‐resolution liquid chromatography–mass spectrometry (LC‐MS) was applied to track the simultaneous incorporation of stable isotope‐labelled and unlabelled substrates from mixed‐sugar mixtures into intracellular metabolites in glycolysis, the PP pathway, ribonucleotide biosynthesis and biofuel precursors. The results unravelled the metabolic hierarchies of glucose with respect to each of the hemicellulosic sugars. Contrary to galactose metabolism, which was subjected to near‐complete inhibition by glucose, mannose was well incorporated into glycolysis as well as biofuel precursors. Investment of both pentoses into ribonucleotide precursors was evident in the presence of glucose, despite the minimal contribution of the pentoses to biofuel precursors via glycolysis. The data also indicated that the PK pathway may connect specifically arabinose to biofuel production, albeit to a relatively smaller contribution compared to glucose. These findings shed light into the constitutive metabolic hierarchy that underpins the channelling of sugar mixtures towards biofuel‐generating pathways in *C. acetobutylicum*.

## Results and discussion

### Proof‐of‐concept labelling experiments

Essential to tracking cellular metabolism is the identification of metabolites in cellular extracts (Fig. 1A). All the relevant metabolites were detected using established methods applying high‐performance LC followed by electrospray ionization and detection using high‐resolution MS in negative mode (Kimball and Rabinowitz, 2006; Rabinowitz and Kimball, 2007; Lu *et al*., 2010; Xu *et al*., 2015; Fig. 1B). The four pentose‐ and hexose‐monophosphates (Xu5P, R5P, G6P, F6P) were detected between retention time (RT) of 7 and 8.5 min and FBP at RT of 13.6 min (Fig. 1B). As a result of the chromatographic separation, the PP metabolites, Xu5P and R5P, were detected despite their common *m/z* value at 229.0120 (Fig. 1B). In a similar fashion, the chromatographic separation allowed for simultaneous detection of G6P and F6P at the same *m/z* channel of 259.0024 (Fig. 1B). The metabolite FBP was detected at *m/z* 338.9887 and chromatographic separation facilitated the detections of both GAP and DHAP at *m/z* 169.9907 (Fig. 1B). It was recently pointed out that, even with soft ionization such as electrospray ionization, in‐source fragmentation can interfere with distinct metabolite detection (Xu *et al*., 2015). One such example is the fragmentation of F6P into GAP (Xu *et al*., 2015) (Fig. 1B). As a result, only the isotopic enrichment of DHAP was monitored here when investigating the metabolism of the different sugar mixtures, which will be discussed in the next sections.

In addition to the phosphorylated metabolites in both the PP and glycolytic pathways, the detection of important precursors to both biofuel and nucleic acid biosynthesis was achieved (Fig. 1A and B). Acetyl‐CoA is a direct precursor to ethanol and, combined with another acetyl‐CoA, to yield acetone as well as butyryl‐CoA, a precursor to butanol (Aristilde *et al*., 2015). At very close RT of 15–15.2 min, acetyl‐coA and butyryl‐CoA were detected, respectively, at *m/z* of 808.1170 and 836.1500 (Fig. 1B). Precursors to *de novo* biosynthesis of purines and pyrimidines, respectively IMP and UMP, were captured at close RT (10.9 and 10.3 min, respectively) but at their distinct *m/z* of 347.0396 and 323.0284 respectively (Fig. 1B). The findings above confirmed that the pertinent metabolites in the metabolic pathways of interest can be detected well by the LC‐MS approach applied here (Fig. 1A and B).

**Figure 2:**
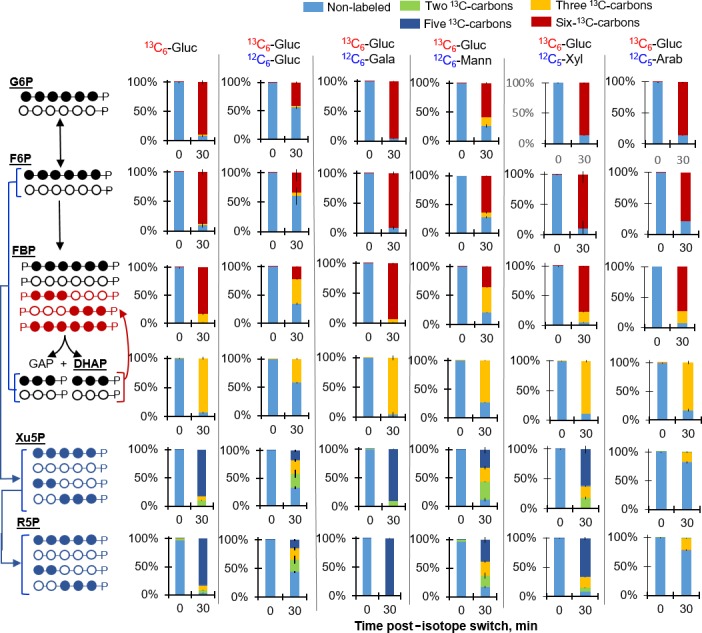
Mixed‐sugar catabolism of stable isotope‐labelled glucose with unlabelled glucose, galactose, mannose, xylose or arabinose. The carbon mapping on the left illustrates the different labeling forms of the metabolites based on isotopic enrichment from substrate feeding. The filled circles and open circles represent, respectively, ^13^C‐labeled carbons and unlabeled carbons; black, red, and blue circles are assigned to labeling schemes in glycolytic, gluconeogenic, and pentose‐phosphate pathways respectively. Labeling of glycolytic and pentose‐phosphate pathway metabolites following 30 min incorporation of fully labelled glucose ([U‐^13^C_6_]‐Gluc) alone or with unlabelled Gluc, galactose (Gala), mannose (Mann), xylose (Xyl) or arabinose (Arab). The carbon mapping on the left illustrates the different labelling forms of the metabolites based on the fed substrate(s). Colour codes for the labelling isotopologues: non‐labelled carbon (light blue), two ^13^C‐carbons (light green), three ^13^C‐carbons (yellow); five ^13^C‐carbons (dark blue), and six ^13^C‐carbons (dark red). The measured data (average ± standard deviation) were from biological replicates (*n *=* *2–3). Non‐noticeable error bars were in cases where standard deviation values were small.

Next, proof‐of‐concept labelling experiments were conducted with *C. acetobutylicum* fed on [U‐^13^C_6_]‐glucose alone or with equimolar unlabelled glucose (Fig. 2). Preliminary kinetic experiments indicated no significant changes in the isotopic enrichment in the glycolytic metabolites after 30 min (Fig. S1). Therefore, only the 30 min labelling data are presented in Fig. 2. When the cells were fed only the labelled glucose, about 90% of both G6P and F6P were fully labelled whereas only up to 40–50% of these metabolites were fully labelled when the cells were fed simultaneously labelled glucose and unlabelled glucose (Fig. 2), in accordance with near equal incorporation of the labelled and unlabelled glucose by 30 min. The slightly less than 50% of fully labelled F6P is due to up to 10% of triply labelled F6P, suggesting gluconeogenic flux from FBP to F6P (Fig. 2). Indeed, the FBP labelling shows clear evidence of gluconeogenic flux (Fig. 2). Forward glycolytic flux would only result in either non‐labelled or fully ^13^C‐labelled FBP but triply ^13^C‐labelled FBP was measured in both glucose labelling schemes, indicating reverse flux of nonlabelled and fully labelled triose‐phosphates (GAP and DHAP) combined to form FBP (Fig. 2). Specifically, 15% and 44% of FBP on average were triply ^13^C‐labelled, respectively, in the cells grown on labelled glucose alone or with unlabelled glucose (Fig. 2). Accordingly, DHAP was found to be over 92% fully labelled in cells fed labelled glucose and over 42% fully labelled in cells fed the mixture of labelled with unlabelled glucose (Fig. 2).

The metabolites (Xu5P and R5P) in the PP pathway also exhibited differences in the labelling, in agreement with the labelling of the hexose‐ and triose‐phosphates as discussed above (Fig. 2 and Fig. S2). When cells were fed only labelled glucose, both Xu5P and R5P were about 82% fully labelled, 6–7% triply ^13^C‐labelled and 5–10% doubly ^13^C‐labelled (Fig. 2); the doubly and triply ^13^C‐labelled are due to reactions involving the minor fractions of nonlabelled hexose‐ and triose‐phosphates remaining at the 30 min labelling time (Figs 2 and S2). Due to higher fraction of nonlabelled hexose‐ and triose‐phosphates in cells grown on both labelled and unlabelled labelled glucose compared to the growth condition with labelled glucose alone, the nonlabelled doubly and triply ^13^C‐labelled fractions for R5P and Xu5P were higher at 31–42%, 20–25% and 19–23% respectively (Figs 2 and S2).

The aforementioned proof‐of‐concept results were used as a guide to determine the hierarchy in the co‐metabolism of glucose with hemicellulosic sugars such that labelling results that match the ^13^C‐glucose‐alone data would indicate complete repression of the accompanying substrate in the presence of glucose whereas labelling results that match the data obtained with the mixture of ^13^C‐glucose and unlabelled glucose would be consistent with equal metabolism of glucose and the hemicellulosic sugar. The following sections detailed the co‐metabolism of labelled glucose with the unlabelled form of each hemicellulosic sugar of interest: galactose, mannose, xylose or arabinose.

### Glucose metabolism with respect to a hemicellulosic hexose: mannose or galactose

Figure 2 also illustrates 30 min labelling patterns of the intracellular metabolites obtained following feeding on [U‐^13^C_6_]‐glucose with an unlabelled hemicellulosic hexose: mannose or galactose (Fig. 2). Kinetic experiments showed that within 15 min, the isotopic enrichment of G6P, F6P and the triose‐phosphate DHAP had reached equilibrium (Fig. S3). Therefore, the 30 min labelling data shown in Fig. 2 represented near steady‐state labelling of these metabolites by the assimilated substrates.

During simultaneous feeding on glucose and galactose, the labelling patterns of metabolites in the glycolytic and PP pathways were about identical to the metabolite labelling during feeding on glucose alone (Fig. 2). These labelling data thus indicated the exclusion of galactose catabolism in the presence of glucose, in agreement with a previous report that glucose was preferred over galactose in *C. acetobutylicum* P262 (Guiterrez and Maddox, 1996). By contrast, the metabolite labelling patterns during simultaneous feeding on glucose and mannose revealed incorporation of both nonlabelled carbons from mannose and the labelled carbons from glucose (Fig. 2). By comparing the specific isotopologues in cells fed ^13^C‐glucose with unlabelled mannose versus those fed ^13^C‐glucose with unlabelled glucose, it was clear that mannose catabolism was not identical to glucose catabolism (Fig. 2).

In the presence of unlabelled mannose and labelled glucose, fully ^13^C‐labelled fractions of G6P, F6P and DHAP were, on average, 58%, 60%, and 73% respectively (Fig. 2). These results implied higher incorporation of glucose than mannose (Fig. 2). The persistence of triply ^13^C‐labelled FBP (on average, 43%) was consistent with the occurrence of gluconeogenic flux in the presence of mannose, similar to the glucose‐only condition (Fig. 2). However, slightly higher fractions of triply ^13^C‐labelled G6P and F6P (on average, up to 15%) implied greater flux of the gluconeogenic flux in the presence of mannose than in the presence of glucose alone (Fig. 2). The presence of partially labelled PP metabolites (Xu5P and R5P) with doubly (up to 24%) and triply (up to 32%) ^13^C‐labelled fractions were also in agreement with the assimilation of mannose in the presence of glucose (Fig. 2). It was previously reported that glucose‐grown *C. cellulotycium*, a less efficient biofuel producer than glucose‐grown *C. acetobutylicum*, exhibited a reversal glycolytic pathway whereby feeding on 50% labeled glucose led to about 12% of triply ^13^C‐labeled F6P (Rabinowitz *et al*., 2015). Therefore, *C. acetobutylicum* grown on a glucose:mannose mixture had a gluconeogenic flux that was comparable to glucose‐grown *C. cellulolitycum*. This gluconeogenic flux was proposed to impair glycolytic flux towards biofuel production in *C. cellulolitycum* (Rabinowitz *et al*., 2015). Whether the same phenomenon can contribute to decreased biosynthesis of biofuel precursors in *C. acetobutylicum* grown on mixed substrates remains to be determined.

**Figure 3:**
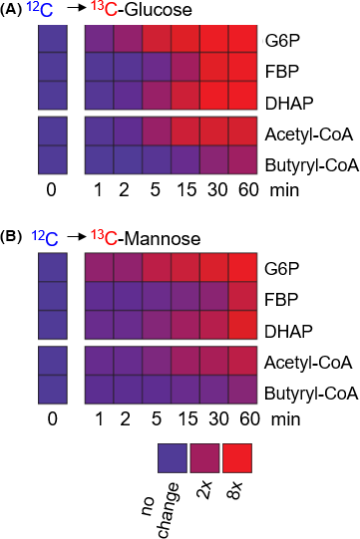
Mannose substitution for glucose in *Clostridium acetobutylicum*. (A) Kinetic metabolite labelling when cells were switched from unlabelled (^12^C) glucose to fully labelled (^13^C) glucose. (B) Kinetic metabolite labelling when cells were switched from unlabelled (^12^C) glucose to fully labelled (^13^C) mannose. Levels of nonlabelled and labelled fractions are shown in blue and red respectively. The measured data used for the heat map are averages from two biological replicates.

A previous study reported similar expression levels of genes encoding for mannose transport and metabolism in glucose‐grown cells versus mannose‐grown cells (Servinsky *et al*., 2010). Therefore, for the cells grown on the glucose:mannose mixture, mannose assimilation may be facilitated by constitutive transporters present in glucose‐grown cells. In fact, it was determined that mannose transport was primarily via glucose transporters in two human cells lines (Rodríguez *et al*., 2005). Here, switch‐substrate labelling experiments demonstrated that *C. acetobutylicum* can fully substitute glucose by rapidly catabolizing mannose (Fig. 3). Within only 1 min following glucose removal and introduction of ^13^C‐labelled mannose, there was already incorporation of mannose‐derived labelled carbons into both glycolytic intermediates and the biofuel precursor acetyl‐CoA (Fig. 3). Within 15 min after the isotope switch, assimilated mannose fully populated the metabolites and, by 60 min, the isotopic enrichment was nearly the same as in cells incorporating labelled glucose (Fig. 3). Therefore, in agreement with results from the transcriptional analysis of glucose‐grown *C*. *acetobutylicum* (Servinsky *et al*., 2010), the results presented here are consistent with constitutive flexibility for mannose uptake and metabolism in glucose‐grown cells.

### Glucose metabolism with respect to a hemicellulosic pentose: xylose or arabinose

In regard to the co‐metabolism of glucose with hemicellulosic pentose sugars in *C*. *acetobutylicum*, Fig. 2 illustrates 30 min labelling of the intracellular metabolites obtained following feeding of *C. acetobutylicum* on [U‐^13^C_6_]‐glucose with unlabelled xylose or unlabelled arabinose. In a previous study (Aristilde *et al*., 2015), data were provided to show that near steady‐state isotopic enrichment in glycolytic and PP intermediates was achieved by 30 min during growth on labelled glucose alone and labelled xylose alone. Here, additional experiments were conducted to confirm that a time period of 30 min was sufficient to achieve near steady‐state isotopic enrichment in the glycolytic and PP pathway metabolites during growth on the glucose:xylose and glucose:arabinose mixtures wherein glucose was fully labelled (Fig. S4). The data indicated no appreciable changes in the labelling patterns of the metabolites extracted at 30 min versus 60 min, thus confirming a 30 min isotopic enrichment was also sufficient (Fig. S4).

The 30 min labelling patterns of glycolytic metabolites indicated that these metabolites were largely exclusively populated by glucose‐derived carbons similar to glucose‐alone conditions (Fig. 2). And, measurements of both non‐labelled and partially labelled Xu5P and R5P compared to the fully labelled PP pathway metabolites measured during growth on labelled glucose alone were consistent with assimilation of the pentose sugars into PP pathway (Fig. 2). These results are thus in agreement with previous results, which showed that the pentose sugars were incorporated into PP pathway metabolites after long‐term isotopic enrichment during cell growth for multiple doubling time on labelled glucose and unlabelled pentose sugar (Aristilde *et al*., 2015). However, the kinetic data obtained here revealed more rapid kinetic incorporation of arabinose than xylose (Fig. 2). Whereas the pentose‐phosphates were about 80% nonlabelled and the remaining fraction triply ^13^C‐labelled in the presence of unlabelled arabinose and labelled glucose, the corresponding labelling pattern in the presence of unlabelled xylose and labelled glucose was, on average, 60–65% quintuply ^13^C‐labelled, 18–20% triply ^13^C‐labelled, and 8–6% doubly ^13^C‐labelled (Fig. 2). This difference indicated that the assimilation of the pentose sugar was more prioritized over the contribution of glucose‐derived carbons for the biosynthesis of the pentose‐phosphates in the presence of arabinose than in the presence of xylose (Fig. 2). These results stressed the preference of arabinose over xylose for uptake and metabolism in the PP pathway, as previously reported (Ezeji and Blaschek, 2007; Aristilde *et al*., 2015).

**Figure 4:**
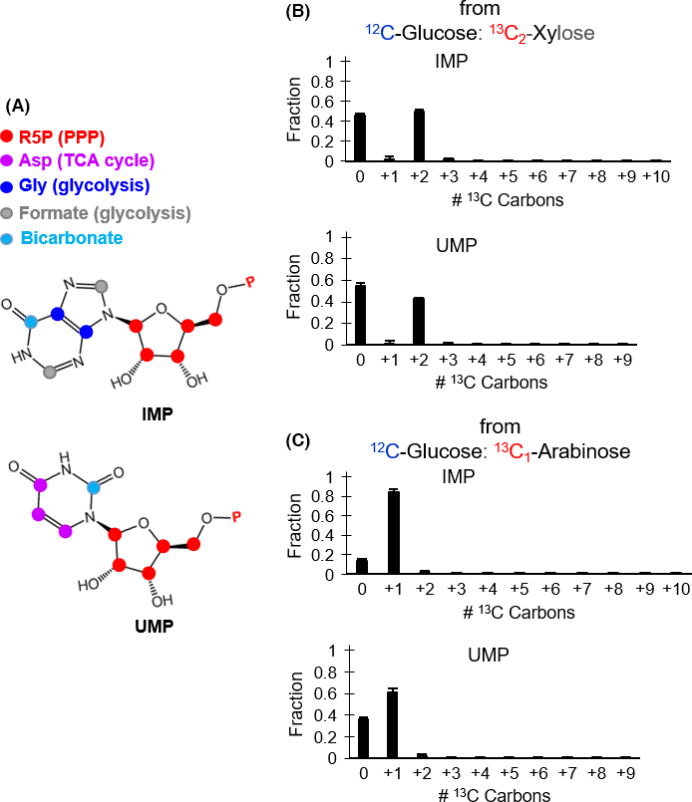
Incorporation of pentoses into the ribonucleotides inosine monophosphate (IMP) and uridine monophosphate (UMP) in the presence of glucose. (A) Metabolic pathways linked to ribonucleotide biosynthesis. (B) Isotopic enrichment of IMP (top) and UMP (bottom) following growth on unlabelled glucose with [1,2‐^13^C_2_]‐xylose. (C) Isotopic enrichment of IMP (top) and UMP (bottom) following growth on unlabelled glucose with [1‐^13^C_1_]‐arabinose. The measured data (average ± standard deviation) were from biological replicates (*n *=* *2–3). Non‐noticeable error bars were in cases where standard deviation values were small.

It was proposed that the assimilation of pentoses into PP pathway in *C. acetobutylicum* with little subsequent contribution towards glycolytic intermediates may be a metabolic strategy to invest pentose‐derived carbons specifically into ribonucleotide biosynthesis (Aristilde *et al*., 2015). The biosynthesis of these ribonucleotides combines metabolites from different metabolic pathways: PPP, glycolysis and TCA cycle (Fig. 4A). The biosynthesis of the purine IMP combines the PP pathway metabolite R5P with glycine [an amino acid derived from the TCA cycle intermediate oxaloacetate (Amador‐Noguez *et al*., 2010)], formate [one‐carbon unit derived from the glycolytic metabolite pyruvate (Amador‐Noguez *et al*., 2010)] and dissolved bicarbonate species (Fig. 4A). And, the biosynthesis of the pyrimidine UMP stems from the combination of R5P with aspartate (a TCA cycle‐derived amino acid) and dissolved carbonate species (Fig. 4A). A preliminary kinetic isotopic flux experiment with fully labelled glucose revealed that the labelling patterns of both IMP and UMP at 60 min were significantly different from those obtained at 30 min (SI, Fig. S5). Therefore, long‐term isotopic enrichment experiments of the ribonucleotides IMP and UMP were conducted wherein the cells were subjected to growth for several hours in minimal medium solution containing unlabelled glucose with either [1,2‐^13^C_2_]‐xylose or [1‐^13^C_1_]‐arabinose in order to evaluate the proposal that assimilated pentoses were routed to ribonucleotide biosynthesis (Fig. 4B and Fig. 4C).

In accordance with the above discussion that glucose dominated glycolysis and downstream metabolic pathways, only R5P from the PP pathway populated by the assimilated pentoses was expected to contribute labelled carbons to the ribonucleotides. Indeed, the labelled forms of both IMP and UMP reflected the labelled forms of R5P under each growth condition: doubly ^13^C‐labelled IMP (50%) and UMP (42%) in cells grown on the glucose:xylose mixture with unlabelled glucose and ^13^C_2_‐xylose; singly ^13^C‐labelled IMP (84%) and UMP (61%) in cells grown on the glucose:arabinose mixture with unlabelled glucose and ^13^C_1_‐arabinose (Fig. 4B and C; SI, Fig. S6). These data also highlight two differences between the two glucose:pentose growth conditions. First, in both conditions, there was a higher isotopic enrichment (by 15–27%, on average) of IMP than of UMP (Fig. 4B and C), implying less *de novo* biosynthesis of the latter than the former. In agreement with more recycling (thus less *de novo* biosynthesis) of UMP than IMP, the kinetic data with fully labelled glucose indicated primarily labelled forms of IMP in accordance with incorporation of glucose‐derived carbons whereas the labelling pattern of UMP still contained, on average, about 34% nonlabelled forms (Fig. S5). Second, there was a higher fraction (by up to 50%, on average) of the labelled forms of both IMP and UMP in the presence of arabinose than xylose (Fig. 4), implying higher rate of *de novo* ribonucleotide biosynthesis in the presence of arabinose. This would be consistent with the aforementioned metabolic preference of arabinose over xylose in *C*. *acetobutylicum* (Aristilde *et al*., 2015).

### Contributions of hemicellulosic sugars to biofuel precursors in the presence of glucose

**Figure 5:**
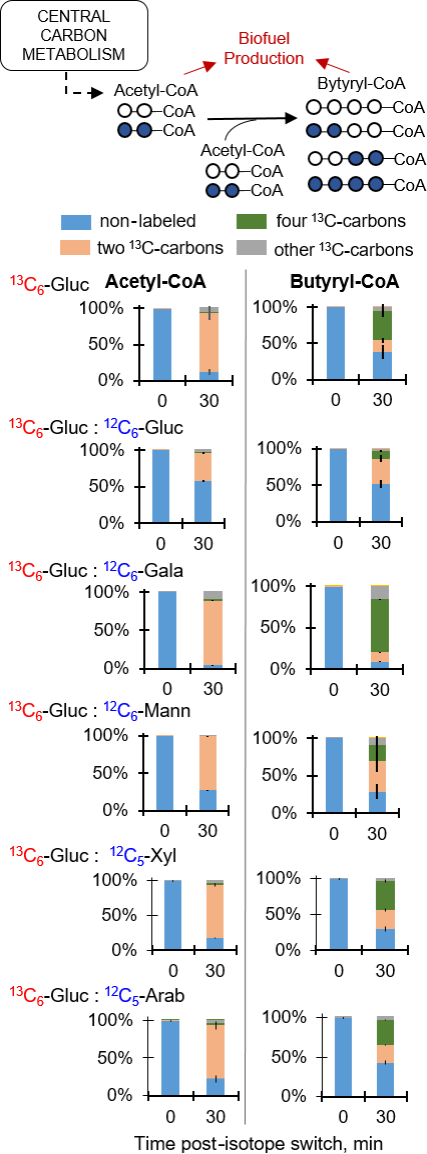
Sugar investment in the biofuel precursors acetyl‐coenzyme A (acetyl‐CoA) and butyryl‐coenzyme A (butyryl‐CoA). Labelling patterns were obtained following 30 min incorporation of substrates as detailed in Fig. 2 legend. The measured data (average ± standard deviation) were from biological replicates (*n *=* *2–3). Non‐noticeable error bars were in cases where standard deviation values were small.

To determine the consequence of the different metabolic hierarchies on the routing of carbons towards biofuel precursors, the labelling patterns of both acetyl‐CoA and butyryl‐CoA were obtained following feeding on fully ^13^C‐labelled glucose alone or with unlabelled glucose, galactose, mannose, xylose or arabinose (Fig. 5). The triose‐phosphates generated in the glycolytic pathway ultimately produce the two‐carbon acetyl moiety in acetyl‐CoA following a decarboxylation step; the butyryl moiety in butyryl‐CoA is the joining of two moles of acetyl moiety (Fig. 5) – the CoA moiety is generated from secondary metabolism that combines ATP with metabolites derived from glycolysis. The short‐term 30 min labelling data obtained here focused on profiling the kinetic labelling of the acetyl and butyryl moieties when labelling of the CoA component would be relatively minor (Fig. 5). Accordingly, during growth on fully labelled glucose alone, acetyl‐CoA was primarily doubly ^13^C‐labelled (on average, greater than 82%) in accordance with the decarboxylation of the triply ^13^C‐labelled fraction (about 90%, on average) of triose‐phosphates as previously described (Fig. 2 and Fig. 5). Interestingly, only about 60% of butyryl‐CoA was labelled with, on average, 17% doubly and 41% quadruply ^13^C‐labelled fractions (Fig. 5). The difference (~20% less) between the ^13^C‐labelled fraction in butyryl‐CoA compared to acetyl‐CoA implied a delay in the metabolic flux to synthesize butyryl‐CoA downstream of acetyl‐CoA (Fig. 5). In the 50:50 mixture with labelled glucose and unlabelled glucose, the labelling pattern of acetyl‐CoA was approaching a near equal fraction of nonlabelled and doubly ^13^C‐labelled fractions, consistent with the incorporation of both fully labelled and nonlabelled glucose‐derived carbons (Fig. 2 and Fig. 5). The subsequent labelling of butyryl‐CoA confirmed the delay in its biosynthetic flux as there was very little quadruply ^13^C‐labelled fraction (Fig. 5).

Next, the contribution of the hemicellulosic hexose sugars to the biofuel precursors in the presence of glucose was determined (Fig. 5). During feeding on labelled glucose and unlabelled galactose, the labelling of acetyl‐CoA, on average at 83% doubly ^13^C‐labelled, was nearly identical to the labelling during feeding on labelled glucose alone, but there was an unexpected higher isotopic enrichment in butyryl‐CoA, with about a 58% increase in the quadruply ^13^C‐labelled fraction when compared to the glucose‐alone condition (Fig. 5). Thus, the labelling patterns of both acetyl‐CoA and butyryl‐CoA during growth on the glucose:galactose mixture were consistent with the complete exclusion of galactose assimilation from the glucose:galactose mixture (Fig. 5). In addition, the higher isotopic enrichment of butyryl‐CoA implied a higher biosynthetic flux of butyryl‐CoA from feeding on the glucose:galactose mixture than feeding on glucose alone (Fig. 5). The significance of this phenomenon warrants further investigation. In terms of mannose contribution to the biofuel precursors during feeding on the glucose:mannose mixture, there was a 12% decrease in the doubly ^13^C‐labelled in acetyl‐CoA and a 50% decrease in the quadruply ^13^C‐labelled in butyryl‐CoA when compared to feeding on glucose alone (Fig. 5). These results indicated that, in contrast to feeding on the glucose:galactose mixture, there was simultaneous routing of carbons from both mannose and glucose towards biofuel precursors during feeding on the glucose:mannose mixture (Fig. 5), athough glucose was still preferred over mannose.

With respect to the pentose contribution to the biofuel precursors in the presence of glucose, the contribution was not the same from xylose and arabinose (Fig. 5). During feeding on the glucose:xylose mixture, the labelling patterns of both acetyl‐CoA and butyryl‐CoA were comparable to those obtained in the presence of glucose alone, indicating little contribution of the xylose assimilated in the PP pathway towards the biofuel precursors (Figs 2 and 5). Accordingly, the reported yields of acids and solvents obtained during *C. acetobutylicum* growth on the glucose:xylose mixture were comparable to those obtained in the presence of glucose alone (Aristilde *et al*., 2015).

Compared to cells grown on the glucose:xylose mixture, the labelling data obtained during feeding on the glucose:arabinose mixture demonstrated an increase in the incorporation of nonlabelled carbons into both acetyl‐coA and butyryl‐CoA by, on average, 28% and 43% greater, respectively (Fig. 5). This investment of arabinose into acetyl‐CoA (Fig. 5), combined with the lack of arabinose‐derived carbons in upper glycolytic metabolites (G6P, F6P, FBP) (Fig. 2), during growth on the glucose:arabinose mixture was in agreement with the previously reported generation of acetyl‐P, a precursor to acetyl‐CoA, equally from glycolysis and the PK pathway (Aristilde *et al*., 2015). It was also shown previously that there was a higher yield (by up to 20%) of acetate following growth on glucose:arabinose compared to growth on glucose:xylose; there was no change, however, in the yield of alcohols and acetone (Aristilde *et al*., 2015). Therefore, the acetyl‐P generated to produce acetyl‐CoA in the presence of arabinose seemed to be discarded as acetate instead of being invested into solvent production (Aristilde *et al*., 2015).

## Concluding remarks

Clostridial species are important in the fermentation of cellulosic and hemicellulosic sugars in environmental matrices and engineered bioreactors. The present study sought to gain metabolic insights into the sugar hierarchies in the notable biofuel producer *C. acetobutylicum* (Rabinowitz *el al*., 2015). The following four hypothesized hierarchies regarding the metabolism of glucose in relation to four different hemicellulosic sugars were evaluated here: (1) glucose inhibition of galactose metabolism, (2) uncompromised co‐metabolism of mannose in the presence of glucose, (3) contribution of pentoses to ribonucleotides and not biofuel production and (4) connection of pentoses to biofuel precursors via the PK pathway. Using ^13^C tracer experiments, intracellular metabolite labelling was monitored to unravel these metabolic hierarchies.

Galactose incorporation into intracellular metabolism was not observed. Thus, the repression of the genes that encode galactose catabolism in glucose‐grown cells (Servinsky *et al*., 2010) persisted during growth on glucose:galactose mixtures. In contrast to galactose metabolism, mannose metabolism was not inhibited by the presence of glucose. Furthermore, it was found that mannose metabolism is analogous to glucose metabolism such that mannose can fully substitute glucose upon glucose absence. Subsequent biochemical studies are needed to determine whether mannose uptake exploits constitutive transporters of glucose in addition to mannose transporters in clostridial species. With respect to the metabolism of the glucose:pentose mixtures, the results revealed that both pentose sugars contributed to *de novo* ribonucleotide biosynthesis. The data were also in agreement with previously reported preference of arabinose over xylose for both consumption (Ezeji and Blaschek, 2007) and assimilation into the PP and PK pathways (Aristilde *et al*., 2015). Moreover, the results here demonstrated appreciable contribution of arabinose to the biofuel precursors via the PK pathway, with potential contribution to acetate but not acetone production according to previous reports of acid and solvent yields from glucose:pentose mixtures (Aristilde *et al*., 2015).

Two important factors should be considered when evaluating the relevance of the metabolic hierarchies presented here for mixed‐sugar utilization in *C. acetobutylicum*. First, plant waste materials are composed of glucose with multiple hemicellulosic sugars simultaneously present. Based on substrate consumption rates during growth of the same strain of *C. acetobutylicum* (strain 824) on a mixture of glucose with multiple hemicellulosic sugars, a previous study (Ezeji and Blaschek, 2007) reported substrate preference of glucose over mannose, glucose over both pentoses, and arabinose over xylose. The last two substrate hierarchies agreed with the metabolomics results presented here, but the first was not consistent with the non‐preferential co‐metabolism of glucose and mannose (Ezeji and Blaschek, 2007). This discrepancy may be due to the 5:1 glucose:mannose ratio in the mixture composition of the previous study (Ezeji and Blaschek, 2007) compared to the 1:1 glucose:mannose mixture used here. It is important to note that the scope of the metabolomics analysis performed here was focused on monitoring glucose metabolism with respect to one hemicellulosic hexose or pentose sugar. Therefore, a metabolomics investigation of *C. acetobutylicum* fed simultaneously on glucose with a complete suite of hemicellulosic sugars is needed to shed light on how the metabolic hierarchies revealed here would manifest in the presence of more complex sugar mixtures.

Second, in order to design optimal engineering strategies for enhancing mixed‐sugar metabolism towards biofuel production, it is important to distinguish between metabolic regulation versus transcriptional regulation (Liao *et al*., 2015; Dash *et al*., 2016; Richter *et al*., 2016). Therefore, as was conducted for *C*. *acetobutylicum* fed on a single hexose or pentose substrate (Servinsky *et al*., 2010), a detailed transcriptional analysis of *C. acetobutylicum* fed on sugar mixtures is warranted. As a necessary complement to this analysis, the present findings provide metabolic evidence for the hierarchical investment of different sugars through central carbon metabolism and towards the biosynthesis of nucleic acids and biofuel precursors.

## Experimental procedures

### Culturing conditions

Batch growth experiments of *C. acetobutylicum* (strain 824, American Type Culture Collection) were conducted in 250 ml Erlenmeyer flasks inside a Bactron IV SHEL LAB (Cornelius, OR, USA) anaerobic chamber (atmosphere: 90% N_2_, 5% H_2_ and 5% CO_2_) at 37°C. An attached sensor continuously monitored the chemical composition of the air inside the chamber. Cells (two to three biological replicates) were grown in a minimal medium solution consisting of 14.7 mM KH_2_PO_4_, 11.5 mM K_2_HPO_4_, 0.81 mM MgSO_4_·7H_2_O, 28.0 mM NH_4_Cl, 1.6 mM CaCl_2_·2H_2_O, 52.7 μM FeSO_4_·7H_2_O, 16.0 nM CuSO_4_·2H_2_O, 0.80 nM MnCl_2_, 1.46 μM CoCl_2_, 0.15 nM Na_2_MoO_4_·2H_2_O, 89.8 nM NiSO_4_·2H_2_O, 0.26 nM ZnCl_2_, 48.5 nM H_3_BO_3_, 532 nM biotin and 1.17 μM 4‐aminobenzoic acid. For the carbon source, the minimal medium was supplemented with a total of 333 mmol C l^−1^ for glucose (i.e., 55.5 mM glucose or 10 g/L glucose) alone or with (at equimolar amount) galactose, mannose, xylose or arabinose. All chemicals were obtained from Fisher or Sigma‐Aldrich (analytical grade). Cell growth was monitored by measuring the optical density at 650 nm (OD_650_).

### Stable isotope tracer experiments

Stable isotope‐labelled sugars were purchased from Cambridge Isotopes (Tewskbury, MA, USA) or Omicron Biochemicals (South Bend, IN, USA). Intracellular kinetic labelling of metabolites in glycolysis, PP pathway, acetyl‐CoA and butyryl‐CoA at each growth condition was conducted following established protocols (Yuan *et al*., 2008; Sasnow *et al*., 2016). Briefly, 3 ml aliquots liquid cultures (three biological replicates) at early exponentially growth phase under each growth condition as described above were filtered (0.45 μm pore size) and the cell‐containing filters were placed on top of plates containing agar‐solidified medium of the same substrate composition. To determine when the cells reached logarithmic growth on the plates, the cells from parallel plates subjected to the same preparation at the same growth condition were rinsed off into a 3 ml suspension for OD_650_ reading. At the early onset of logarithmic growth, the filters containing the cells were switched from the unlabelled media plates to media plates with fully labelled glucose ([U‐^13^C_6_]‐glucose) combined with either unlabelled glucose or the hemicellulosic sugar. Therefore, the experiments with isotopic switch were the following: from unlabelled glucose to labelled glucose, from unlabelled glucose to 1:1 labelled glucose:unlabelled glucose, from 1:1 unlabelled glucose:unlabelled galactose to 1:1 labelled glucose:unlabelled galactose, from 1:1 unlabelled glucose:unlabelled mannose to 1:1 labelled glucose:unlabelled mannose, from 1:1 unlabelled glucose:unlabelled xylose to 1:1 labelled glucose:unlabelled xylose, and from 1:1 unlabelled glucose:unlabelled arabinose to 1:1 labelled glucose:unlabelled arabinose. Metabolism was quenched (see details in the next section) after specific time points: 1, 2, 5, 15, 30 or 60 min. Cells that were only grown on unlabelled media were used as a control for time 0 min.

Faster rate of isotopic enrichment was found for central carbon metabolites than for ribonucleotides during kinetic isotopic enrichment with fully labelled glucose (Fig. S5). Therefore, to monitor incorporation of pentoses into ribonucleotides during growth on the glucose:pentose mixtures, long‐term isotopic enrichment experiments were performed using liquid cultures (three biological replicates) grown for at least two doubling times on unlabelled glucose with either doubly ^13^C‐labelled xylose ([1,2‐^13^C_2_]‐xylose) or singly ^13^C‐labelled arabinose ([1‐^13^C_1_]‐arabinose).

### Monitoring intracellular metabolite labelling

Cellular metabolism for each of the tracer experiments described above (two to three biological replicates) was quenched by quickly submerging cell‐containing filters from media plates or filtered cells from the liquid cultures into a cold (−20°C) solvent mixture composed of 40:40:20 methanol:acetonitrile:water as previously described (Kimball and Rabinowitz, 2006; Sasnow *et al*., 2016). Metabolites were isolated by reverse‐phase high‐performance LC with high‐accurate orbitrap MS operated in negative mode on a Thermo Exactive mass spectrometer following established methods (Lu *et al*., 2010; Xu *et al*., 2015). Using standards, the detection of the following metabolites was verified: R5P, Xu5P, G6P, F6P, FBP, IMP, UMP, acetyl‐CoA, butyryl‐CoA. Using the MAVEN software package (Clasquin *et al*., 2012), the multiple isotopologues (different labelled forms of the same compound with the same number of ^13^C‐labelled carbons) resulting from the stable isotope tracer experiments were determined. The ^13^C‐labelled fractions were corrected for the natural abundance of ^13^C.

## Conflict of Interest

None declared.

## Supporting information


**Fig. S1.** Proof‐of‐concept labelling kinetics of glycolytic metabolites during feeding on glucose.
**Fig. S2.** Carbon mapping of glycolytic metabolites connecting to pentose‐phosphate pathway intermediates via a transketolase reaction.
**Fig. S3.** Kinetic incorporation of glucose in the presence of hemicellulosic hexose sugars.
**Fig. S4.** Kinetic incorporation of glucose in the presence of hemicellulosic pentose sugars.
**Fig. S5.** Kinetic isotopic enrichment of metabolite precursors to ribonucleotide biosynthesis during feeding on fully labelled glucose.
**Fig. S6.** Long‐term pentose assimilation into ribose‐5‐posphate during feeding on glucose:pentose mixtures.
