# Supplementary material for: Metabolite labelling reveals hierarchies in Clostridium acetobutylicum that selectively channel carbons from sugar mixtures towards biofuel precursors
Source: Microb Biotechnol. 2016 Nov 22;10(1):162–74. doi: 10.1111/1751-7915.12459 (PMC5270725; doi:10.1111/1751-7915.12459)
Supplement: Supplementary file 1 — Fig. S1. Proof‐of‐concept labelling kinetics of glycolytic metabolites during feeding on glucose. Fig. S2. Carbon mapping of glycolytic metabolites connecting to pentose‐phosphate pathway intermediates via a transketolase reaction. Fig. S3. Kinetic incorporation of glucose in the presence of hemicellulosic hexose sugars. Fig. S4. Kinetic incorporation of glucose in the presence of hemicellulosic pentose sugars. Fig. S5. Kinetic isotopic enrichment of metabolite precursors to ribonucleotide biosynthesis during feeding on fully labelled glucose. Fig. S6. Long‐term pentose assimilation into ribose‐5‐posphate during feeding on glucose:pentose mixtures. [file MBT2-10-162-s001.pdf]

Metabolite labelling reveals hierarchies in *Clostridium acetobutylicum* that selectively channel carbons from sugar mixtures towards biofuel precursors

Ludmilla Aristilde*,†

†Department of Biological and Environmental Engineering, College of Agriculture and Life Sciences, Cornell University, Ithaca, NY 14853, USA,

*Corresponding Author:

Phone: (607) 255-6845. Fax: (607) 255-4449. E-mail: ludmilla@cornell.edu

SUPPLEMENTARY INFORMATION

Fig. S1. Proof-of-concept labeling kinetics of glycolytic metabolites *C. acetobutylicum* during feeding on glucose

Fig. S2. Carbon mapping of glycolytic metabolites connecting to pentose-phosphate pathway intermediates via a transketolase reaction.

Fig. S3. Kinetic incorporation of glucose in the presence of hemicellulosic hexose sugars in *C. acetobutylicum.*

Fig. S4. Metabolite precursors to biosynthesis of ribonucleotides.

Fig. S5. Kinetic isotopic enrichment of metabolite precursors to ribonucleotide biosynthesis during feeding on fully-labeled glucose.

Fig. S6. Long-term pentose assimilation into ribose-5-posphate during feeding on glucose:pentose mixtures.

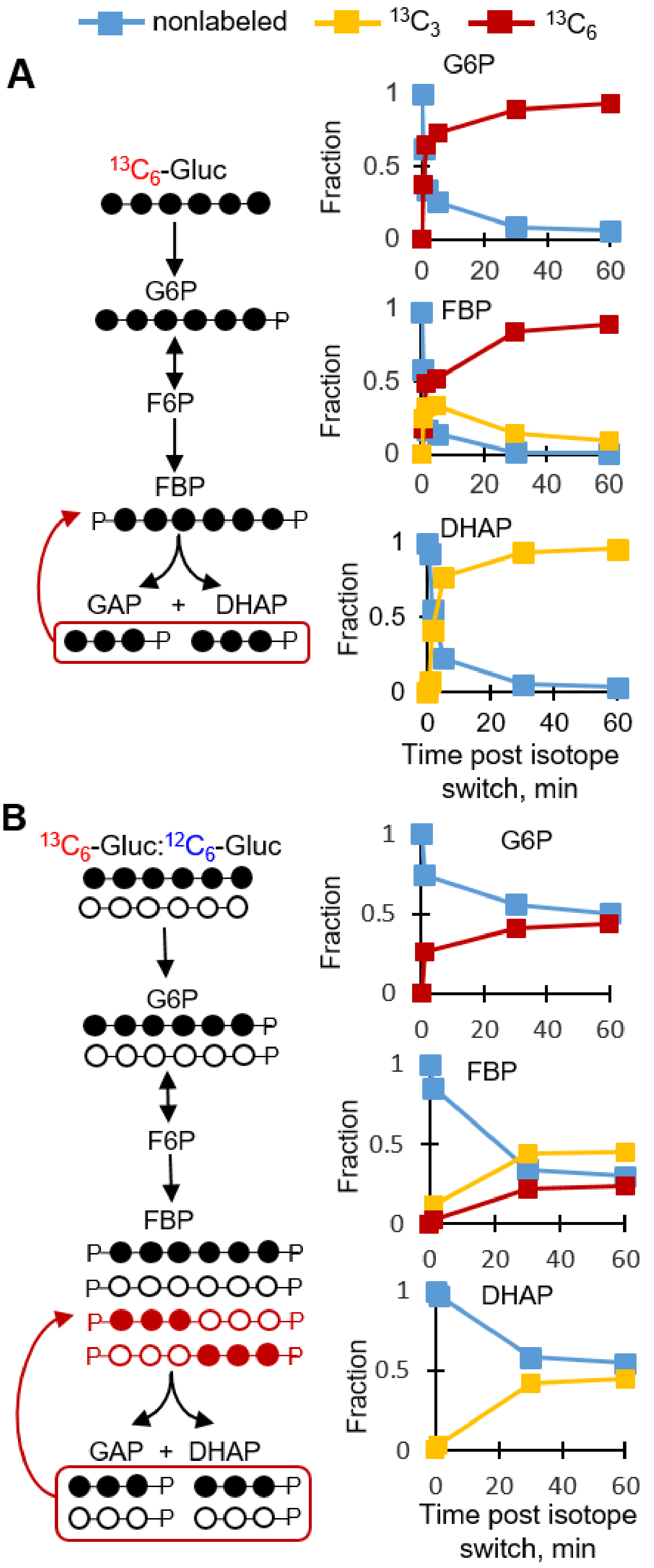

Fig. S1. Proof-of-concept labeling kinetics of glycolytic metabolites *C. acetobutylicum* during feeding on glucose. (A) Carbon mapping (left) and intracellular labeling kinetics data (right) during feeding on fully-labeled glucose ([U-$^{13}C_6$]-glucose). (B) Carbon mapping (left) and intracellular labeling kinetics data (right) during feeding on fully-labeled glucose ([U-$^{13}C_6$]-glucose) and nonlabeled glucose (^{12}C-glucose). Abbreviations: glucose-6-phosphate, G6P; fructose-1,6-bisphosphate, FBP; drihydroxyacetone-3-phosphate, DHAP. The measured data (average ± standard deviation) were from biological replicates (*n* =2-3). The error bars were minimal (less than 5%) and are not visible in the graphs.

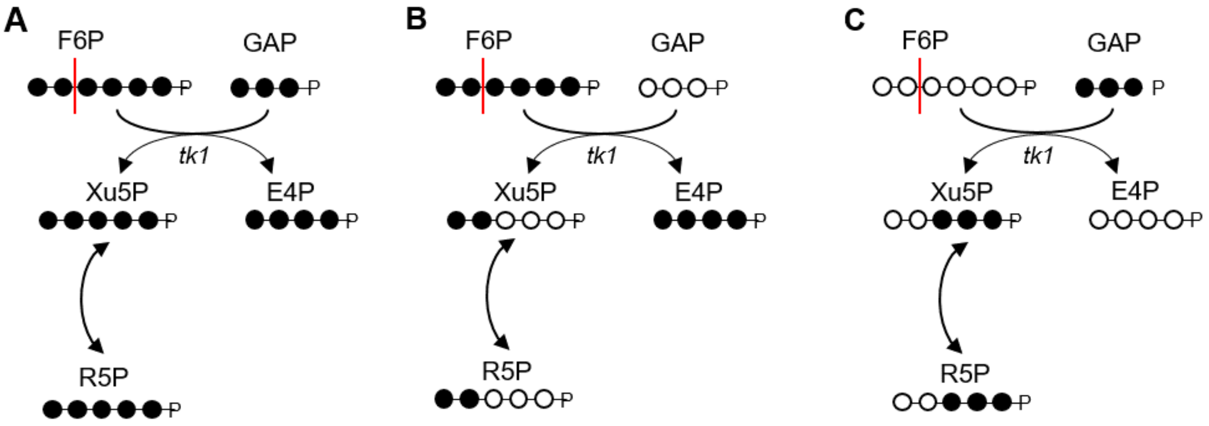

Fig. S2. Carbon mapping of glycolytic metabolites connecting to pentose-phosphate pathway intermediates via a transketolase reaction (*tk1*). (A) Carbon mapping with fully-labeled glycolytic metabolites as reactants. (B and C) carbon mapping with a combination of full-labeled and non-labeled glycolytic metabolites as reactants. Abbreviations: fructose-1,6-bisphosphate, FBP; glyceraldehyde-3-phosphate, GAP; xylulose-5-phosphate, Xu5P; ribose-5-phosphate, R5P; erythrose-4-phosphate.

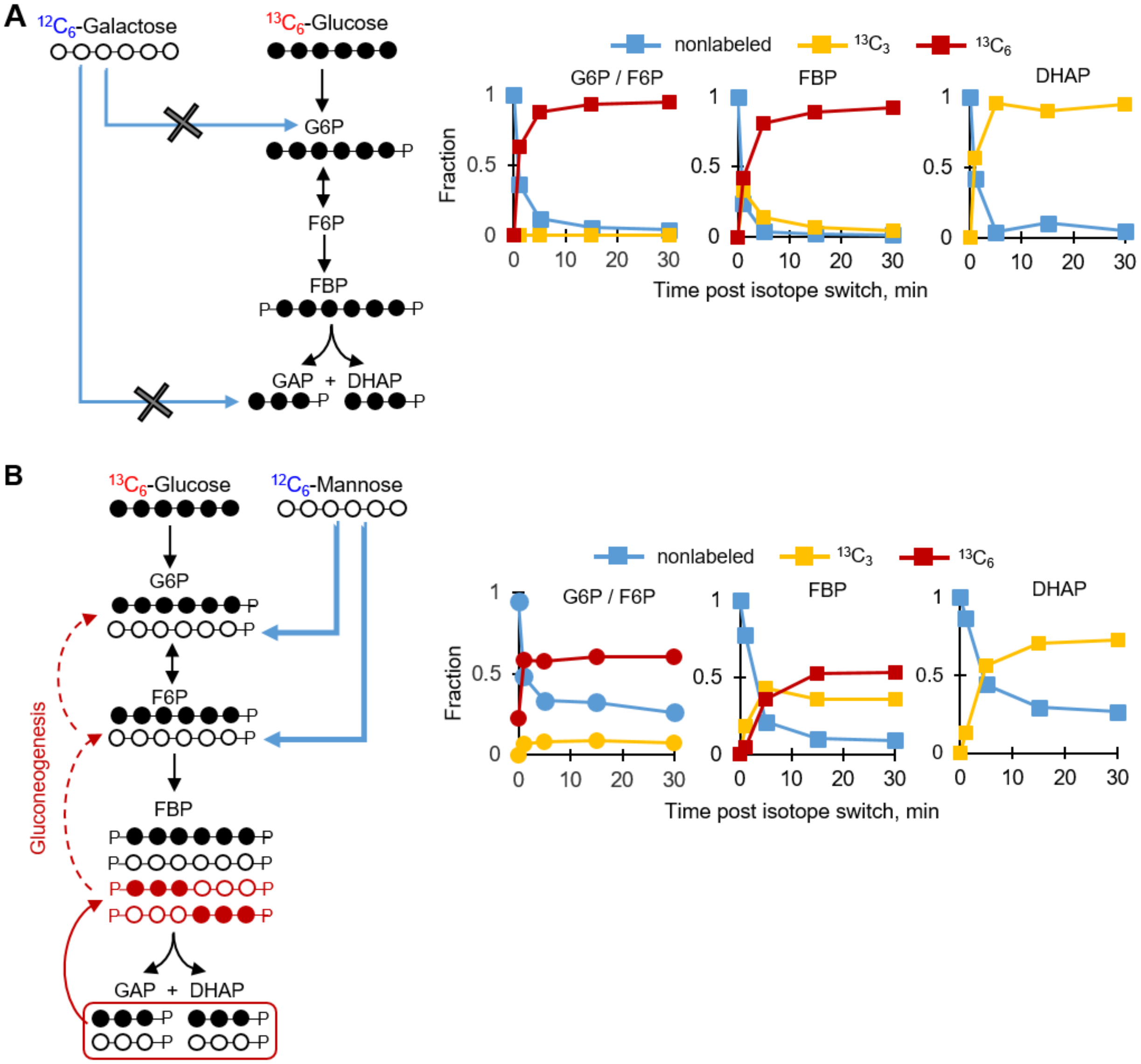

Fig. S3. Kinetic incorporation of glucose in the presence of hemicellulosic hexose sugars in *C. acetobutylicum.* (A) Carbon mapping (left) and intracellular labeling kinetics data (right) during feeding on fully-labeled glucose ([U-$^{13}C_6$]-glucose) and nonlabeled galactose (^{12}C-galactose). (B) Carbon mapping (left) and intracellular labeling kinetics data (right) during feeding on fully-labeled glucose ([U-$^{13}C_6$]-glucose) and nonlabeled mannose (^{12}C-mannose). Abbreviations: glucose-6-phosphate, G6P; fructose-1,6-bisphosphate, FBP; drihydroxyacetone-3-phosphate, DHAP. The measured data (average ± standard deviation) were from biological replicates (*n* =2). The error bars were minimal (less than 3%) and are not visible in the graphs.

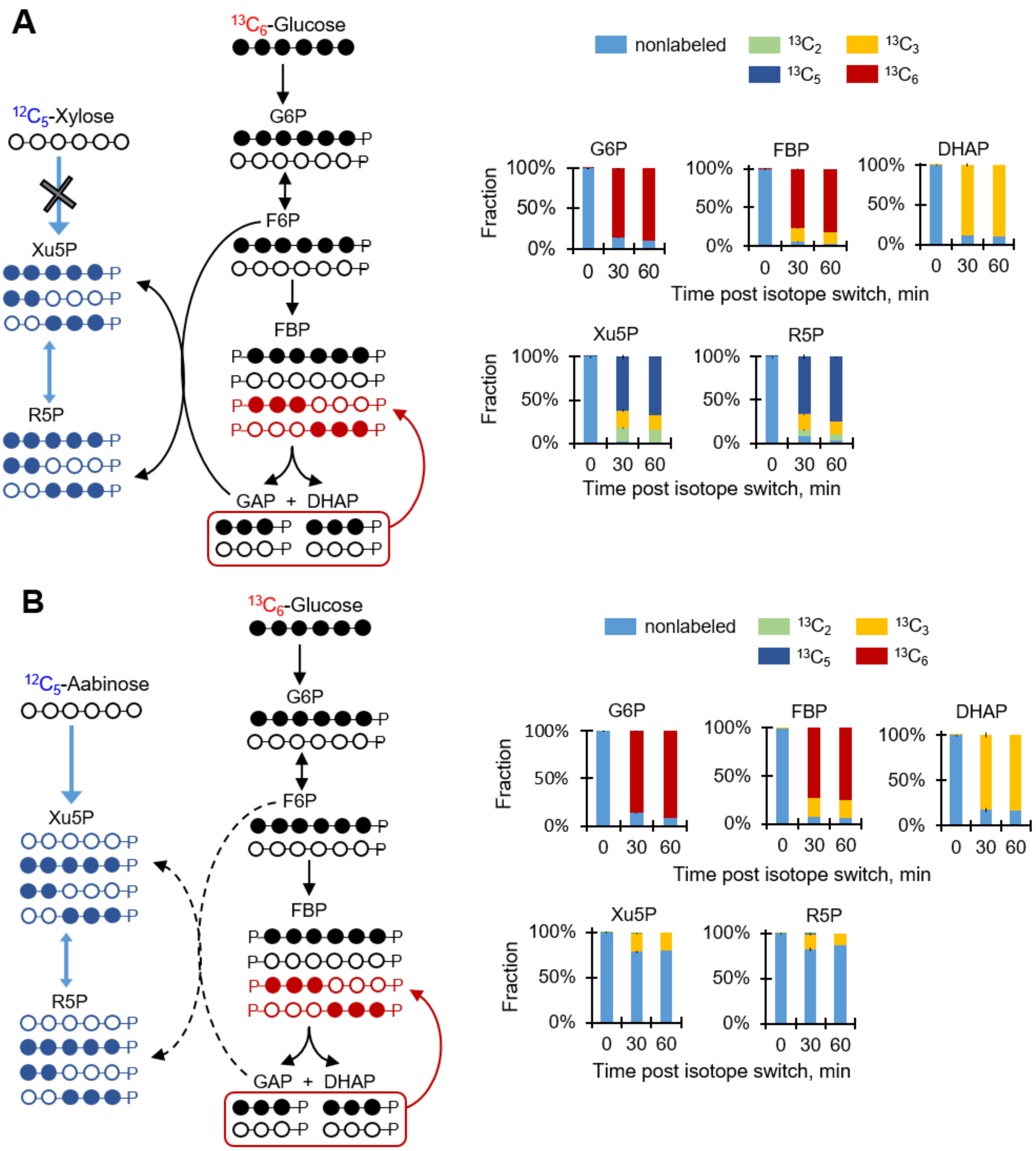

Fig. S4. Kinetic incorporation of glucose in the presence of hemicellulosic pentose sugars in *C. acetobutylicum.* (A) Carbon mapping (left) and intracellular labeling kinetics data (right) during feeding on fully-labeled glucose ([U-$^{13}C_6$]-glucose) and nonlabeled xylose ($^{12}C_5$-xylose).
(B) Carbon mapping (left) and intracellular labeling kinetics data (right) during feeding on fully-labeled glucose ([U-$^{13}C_6$]-glucose) and nonlabeled arabinose ($^{12}C_5$-arabinosee). Abbreviations: xylulose-5-phosphate, Xu5P; ribose-5-phosphate, R5P; glucose-6-phosphate, G6P; fructose-1,6-bisphosphate, FBP; drihydroxyacetone-3-phosphate, DHAP. The measured data (average ± standard deviation) were from biological replicates (*n* =2). The error bars were minimal (less than 6%) and are thus barely visible in the graphs.

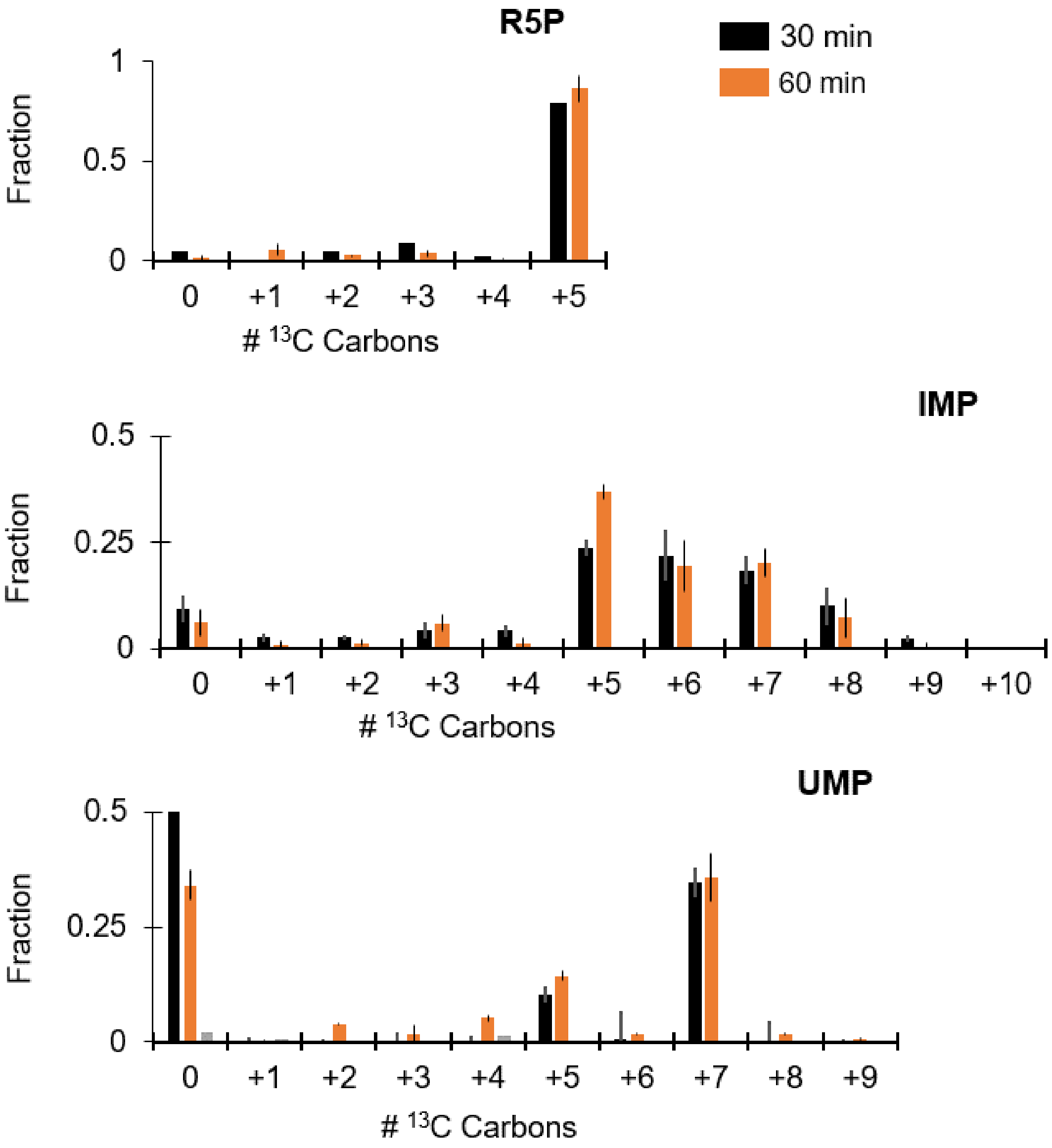

Fig. S5. Kinetic isotopic enrichment of metabolite precursors to ribonucleotide biosynthesis during feeding on fully-labeled glucose. Metabolite labeling pattern of ribose-5-phosphate (R5P), inosine monophosphate (IMP), and uridine monophosphate (UMP) after 30 min (black bars) and 60 min (orange bars) after isotopic switch from unlabeled glucose to labeled glucose. The measured data (average ± standard deviation) were from biological replicates (n = 3).

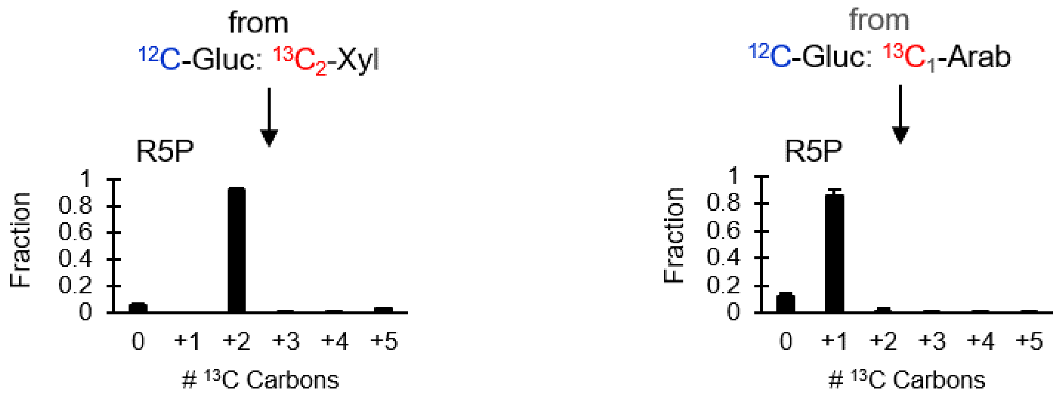

Fig. S6. Long-term pentose assimilation into ribose-5-posphate (R5P) in the presence of glucose. Long-term isotopic enrichment of R5P in *C. acetobutylicum* during growth on nonlabeled (^{12}C-Gluc) and two-carbon labeled xylose ([1,2-$^{13}C_2$]-Xyl) or unlabeled (^{12}C-Gluc) and one-carbon labeled arabinose ([1-$^{13}C_1$]-Arab). The measured data (average ± standard deviation) were from biological replicates (*n* = *3*).
